# P-59. Clinical predictors of ICU admission in patients with invasive Pneumococcal blood stream infection: a review of 10-year single-center experience

**DOI:** 10.1093/ofid/ofaf695.288

**Published:** 2026-01-11

**Authors:** Ayesha Samreen, Rita Igwilo-Alaneme, Nischal Ranganath, Omar M Abu Saleh

**Affiliations:** Mayo Clinic, Rochester, MN; Mayo Clinic, Rochester, MN; Mayo Clinic, Rochester, MN; Mayo Clinic, Rochester, MN

## Abstract

**Background:**

Despite advancements in vaccination, mortality from Pneumococcal blood stream infections remains high (∼20%). Risk factors include host comorbidities (e.g., immunosuppression, kidney disease, alcohol use, smoking) and certain serotypes. While intensive care unit (ICU) admission and septic shock are associated with poor outcomes, predictors of severe disease remain underexplored.

**Figure:**
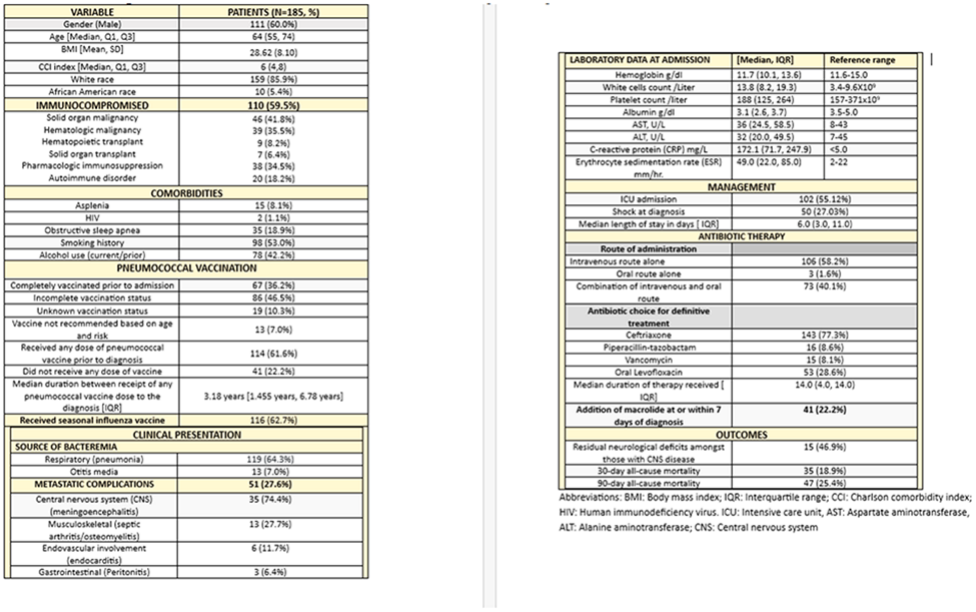
Characteristics of patients with ICU admission within 24 hours of invasive pneumococcal disease diagnosis Epidemiology, clinical characteristics, metastatic complications and management of invasive pneumococcal disease (IPD)

**Figure:**
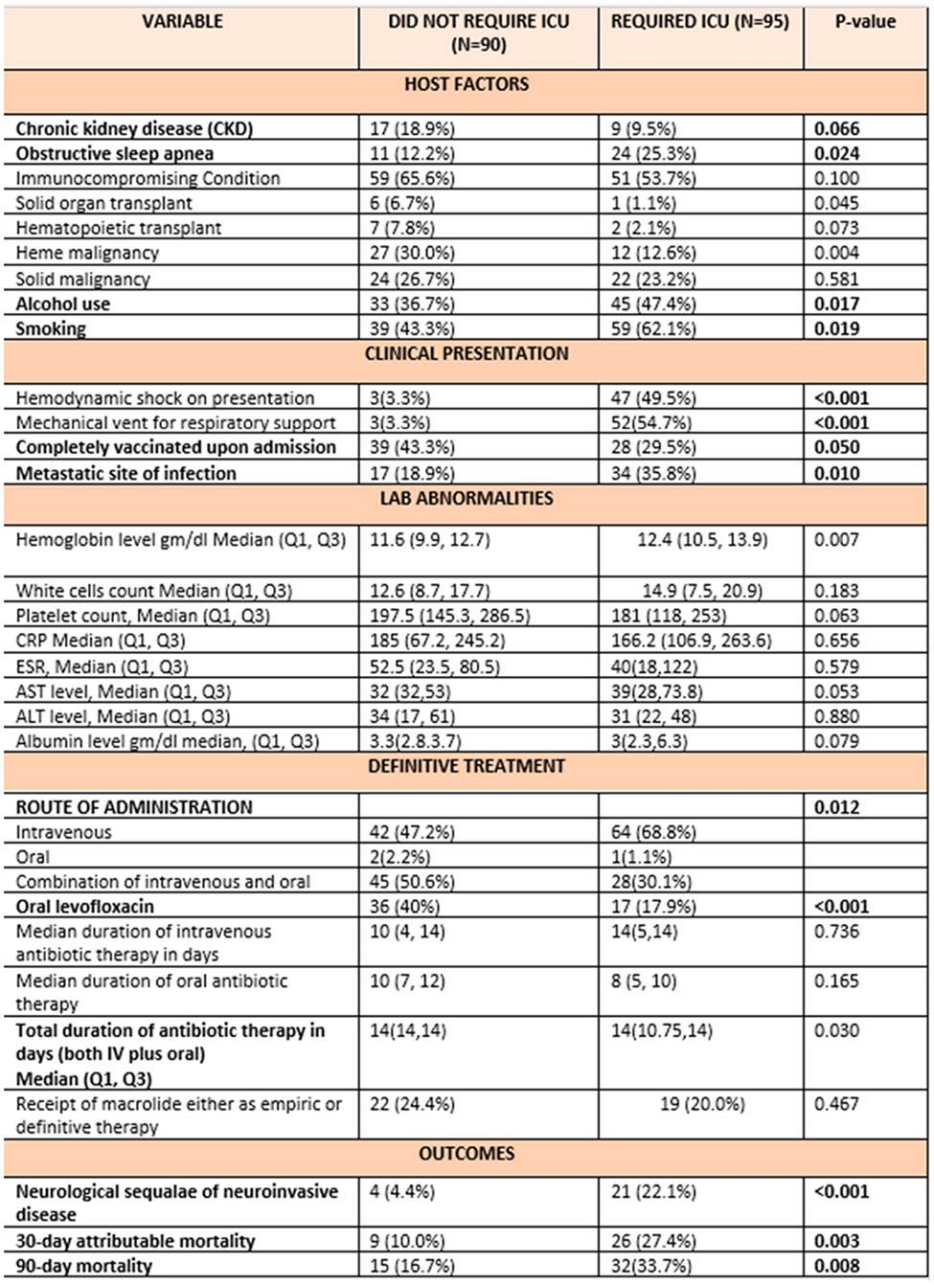

**Methods:**

We conducted a retrospective review of hospitalized adults with *Streptococcus pneumoniae* bacteremia between 2013-2023. Clinical comorbidities, laboratory values, vaccination status, microbiologic data, and treatment strategies were collected. Descriptive statistics and univariate logistic regression were used to assess predictors of ICU admission using R Studio v4.4.3

**Figure:**
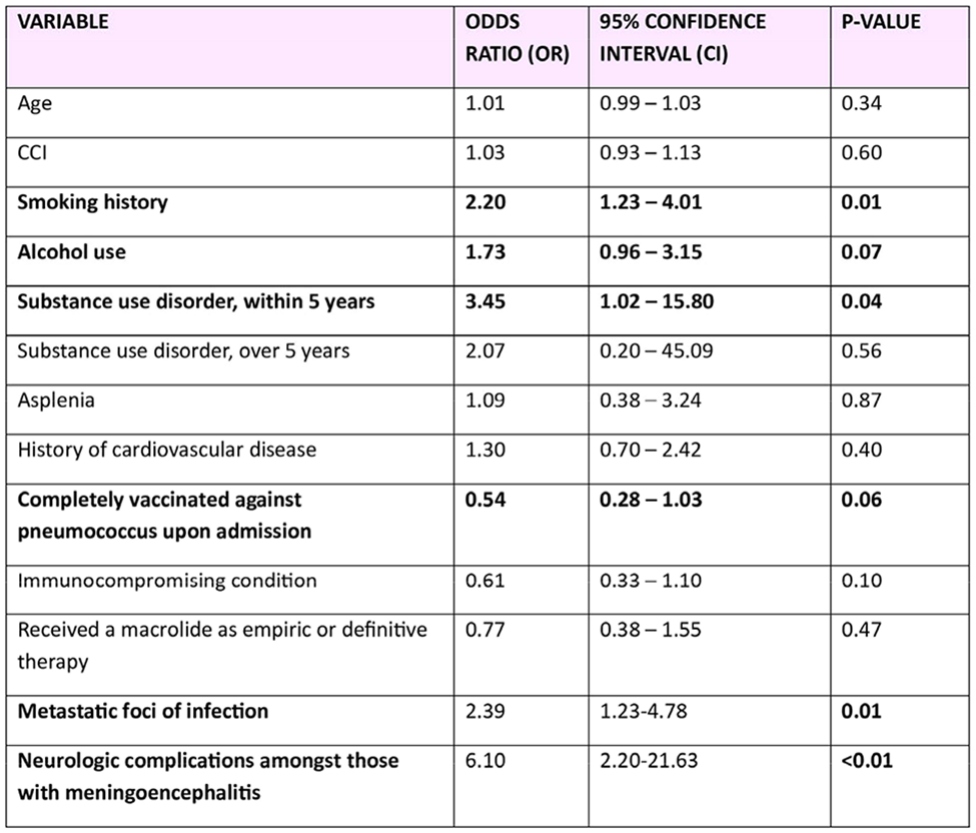
Univariate logistic regression analysis for predictors of ICU admission within 24 hours of diagnosis of streptococcus pneumoniae blood stream infection Rates of streptococcus pneumoniae blood cultures over the years- seasonality trends

**Figure:**
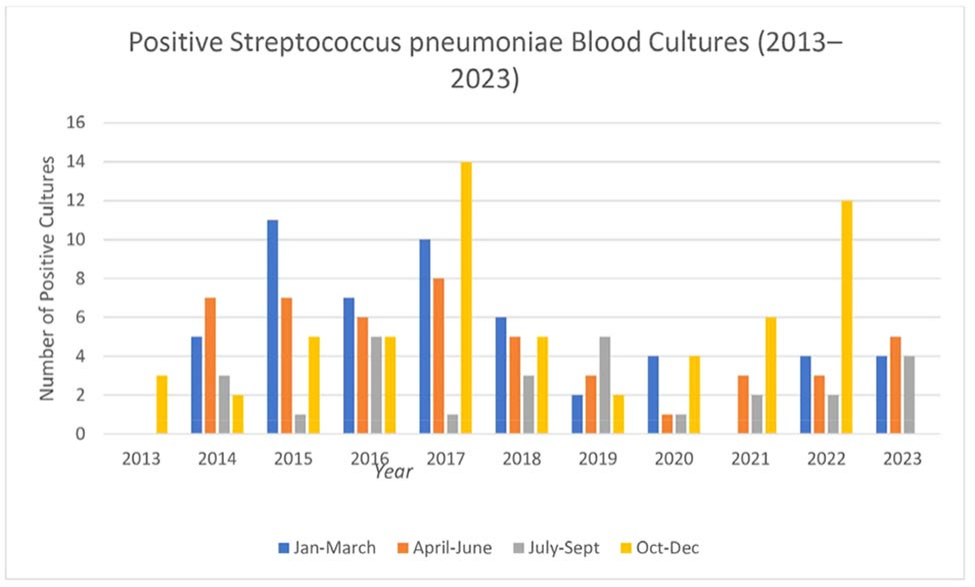

**Results:**

A total of 185 patients were included; the majority were male (60%) with a median age of 64 years (Table 1). Immunocompromising conditions were present in 60%, and only 43% were up to date with pneumococcal vaccination. Metastatic infection was identified in 28% of cases, most commonly meningoencephalitis (74%), musculoskeletal involvement (28%), and endocarditis (12%). ICU admission within 24 hours of presentation was required in 55% of patients (Tables 2 and 3).

Predictors of ICU admission included active smoking (OR 2.20), substance use within the past five years (OR 3.45), presence of metastatic foci (OR 2.39), and neurologic complications (OR 6.10). Up-to-date pneumococcal vaccination demonstrated a trend toward a protective effect. 30-day and 90-day mortality rates were 18.9% and 25.4%, respectively. Other predictors of 30-day mortality included smoking history, higher Charlson comorbidity index (CCI), hemodynamic shock at admission, and lab abnormalities such as hypoalbuminemia and elevated liver enzymes.

Among the 69 isolates with available serotype data, serogroups 22 and 15 were most frequently identified.

**Conclusion:**

Pneumococcal bloodstream infections are associated with substantial mortality, particularly among patients requiring ICU admission. Smoking, recent substance use, and the presence of metastatic complications were significantly associated with severe disease and ICU admission. Up-to-date pneumococcal vaccination demonstrated a protective effect.

**Disclosures:**

All Authors: No reported disclosures

